# Editorial: Gut feelings: investigating the link between microbiota and kidney disease progression

**DOI:** 10.3389/fimmu.2025.1579198

**Published:** 2025-03-11

**Authors:** Marijn M. Speeckaert, Laureline Berthelot

**Affiliations:** ^1^ Department of Nephrology, Ghent University Hospital, Ghent, Belgium; ^2^ Research Foundation-Flanders (FWO), Brussels, Belgium; ^3^ Center of Research in Transplantation and Translational Immunology CR2TI, UMR1064, INSERM, Nantes University, Nantes, France

**Keywords:** gut-kidney axis, microbiota dysbiosis, chronic kidney disease, inflammation, metabolites, microbiometargeted therapy

In recent years, researchers have focused increasingly on the delicate relationship between gut microbiota and kidney function, showing a complex network of interactions that influence kidney disease progression (1–4). The gut-kidney axis, a bidirectional communication pathway that connects gut bacteria to kidney function, is essential for metabolic regulation, immune responses, and inflammatory processes. Disruptions in this system, known as dysbiosis, contribute to kidney disease by activating inflammatory cascades, changing metabolite synthesis, and affecting immunological homeostasis. Studies (5–7) suggest that the gut microbiota not only contributes to kidney disease progression, but also influences therapy efficacy. Regulating particular bacterial strains with diet and tailored probiotics may improve kidney function and halt disease progression. Additionally, it is now known that microbial metabolites such uremic toxins and short-chain fatty acids play a significant role in kidney health. Through the regulation of metabolites, interventions targeting the gut microbiota have the potential to reduce oxidative stress and inflammation in patients with chronic kidney disease (CKD). Furthermore, the composition of the gut microbiota can be used to diagnose early-stage kidney disease, allowing more prompt treatment. This rising body of research emphasizes the need of incorporating microbiome analysis into nephrology practices, which could revolutionize how kidney diseases will be detected and handled in the future.

In their study on IgA nephropathy (IgAN), a prevalent form of CKD, Liu et al. highlight the role of gut microbiota in modifying immune responses. The study finds that kidney inflammation is caused by galactose-deficient IgA1 buildup, which is facilitated by microbial imbalances. Their findings indicate that gut-targeted therapy, such as probiotics and dietary changes, might be able to restore microbial balance and delay the onset of IgAN. Crucially, this study illustrated the broader implications of immune regulation via microbiota, linking kidney protection to gut health. Liu et al. also presented the gut-lung-kidney axis, arguing that gut dysbiosis impacts both kidney and pulmonary functions. This study demonstrates that microbial translocation and airborne contaminants cause systemic inflammation, which promotes CKD. Their findings stress the importance of environmental influences on kidney function and microbiota composition, and they advocate for a multidisciplinary approach to treatment strategies that account for both external and microbial effects. Yao et al. expand on the issue of IgAN by providing a complete assessment of how gut microbiota promotes development and progression of disease. They focused on three main mechanisms: intestinal barrier breakdown, alterations in microbial metabolites, and aberrant mucosal immune responses. Changes in the gut microbiota might enhance intestinal permeability, allowing inflammatory chemicals to flow throughout the body and worsen kidney disease. Yao et al. studied therapy approaches such as probiotics, fecal microbiota transplantation, antibiotics, herbal medicine (Zhen Wu Tang), gluten-free diets, and hydroxychloroquine therapies in attempt to transform gut microbiota and improve IgAN outcomes. Their findings strongly imply that microbiota modulation is one of the most essential strategies for treating IgAN.

Complexity of interactions between the host and its microbiota can be assessed by genetics. Feng et al. conduct a Mendelian randomization research and find genetic evidence supporting the kidney-microbiota relationship. By identifying specific bacterial species associated with glomerulonephritis, chronic tubulointerstitial nephritis, and nephrotic syndrome, their research establishes a link between microbial alterations and kidney diseases. These findings illustrate the potential of microbiome-based precision medicine, which uses genetic indicators of microbial imbalance to predict kidney disease and steer specific treatments.

Ren et al. investigate the importance of systemic inflammation in the evolution of kidney disease. Their research reveals the significance of gut microbiota in modifying inflammatory pathways, distinguishing between protective bacterial strains that produce short-chain fatty acids and pathogenic bacteria that cause inflammation via endotoxin release. By highlighting the delicate balance between beneficial and harmful microbial populations, their work strengthens the case for microbiota-targeted therapies to mitigate chronic inflammation in CKD patients.

Another important factor in kidney disease progression is metabolic dysfunction, particularly in the context of diabetes. Yan et al. investigate the relationship between gut microbiota and diabetic nephropathy (DN), finding that changes in microbial composition lead to glucose metabolism dysregulation and renal inflammation. Their research finds specific bacterial strains, such as Verrucomicrobiae, that are associated with higher DN risk, whereas others, such as Bifidobacterium, have protective effects. This study emphasizes the potential for microbiome-modulating strategies to supplement current diabetic treatments and lower the risk of kidney problems.

Zhang et al. investigate the effect of *Bifidobacterium bifidum* supplementation on diabetic renal impairment. Clinical studies show that ingesting probiotics can strengthen the intestinal barrier, reduce systemic inflammation, and improve metabolic indicators. Wang et al. study how traditional herbal formulations such as Jin Gui Ren Qi Pill and *Clerodendranthus* sp*icatus* affect intestinal flora. Their findings support the use of natural medicines to treat kidney disease by demonstrating that herbal therapies reduce inflammatory bacteria species while enhancing beneficial bacterial populations.

Wang et al. broaden the scope of microbiome study to include hyperuricemic nephropathy. Their findings show that certain bacterial strains alter uric acid metabolism, which contributes to oxidative stress and kidney inflammation. By identifying microbial targets important in uric acid control, they propose a novel therapeutic method that uses probiotics to improve uric acid excretion and reduce kidney injury.

On a bigger scale, Chen et al.’s epidemiological study underscores the widespread impact of microbiota imbalances on CKD. Their large-scale study in Mianzhu, China, compared gut microbiota patterns with CKD prevalence, demonstrating links between microbial composition, obesity, diabetes, and hypertension. These findings underscore the importance of incorporating microbiome screening into normal clinical practices in order to identify high-risk individuals for kidney disease and develop preventive interventions.

These findings suggest that gut bacteria have a substantial role in kidney disease. An increasing amount of evidence suggests that microbial imbalances are not only coincidental, but also substantial causes of kidney failure. Understanding how gut bacteria interact with immunological, metabolic, and inflammatory processes opens the door to new, non-invasive treatment options. The future of kidney disease treatment may lay in microbiome-targeted therapeutics, which range from dietary interventions and probiotics to precision medicine techniques based on genetic microbiota profiles.

Future research should focus on creating drugs based on microbiomes, using clinical and genetic data to improve treatment outcomes. Combining microbiome and nephrology research could result in new, more comprehensive approaches for protecting kidney function.
